# The effect of red grape seed extract on serum paraoxonase activity in patients with mild to moderate hyperlipidemia: O efeito do extrato de semente de uva vermelha na atividade do soro paraoxonase em doentes com hiperlipidemia leve a moderada

**DOI:** 10.1590/1516-3180.2015.01702312

**Published:** 2016-05-13

**Authors:** Hassan Argani, Amir Ghorbanihaghjo, Hamid Vatankhahan, Nadereh Rashtchizadeh, Sina Raeisi, Hadi Ilghami

**Affiliations:** I MD. Professor, Drug Applied Research Center, Tabriz University of Medical Sciences, Tabriz, Iran.; II MSc, PhD. Professor, Drug Applied Research Center, Tabriz University of Medical Sciences, Tabriz, Iran.; III MSc. Drug Applied Research Center, Tabriz University of Medical Sciences, Tabriz, Iran.; IV MSc, PhD. Professor, Biotechnology Research Center, Tabriz University of Medical Sciences, Tabriz, Iran.; V MSc. Doctoral Student, Biotechnology Research Center, Tabriz University of Medical Sciences, Tabriz, Iran.; VI MSc. Doctoral Student, Faculty of Medicine, Tabriz University of Medical Sciences, Tabriz, Iran.

**Keywords:** Cholesterol, HDL, Apolipoprotein A-I, Flavonoids, Cholesterol, LDL, Cholesterol

## Abstract

**CONTEXT AND OBJECTIVE:**

Red grape seed extract (RGSE) contains oligomeric proanthocyanidin complexes as a class of flavonoids. These compounds are potent antioxidants and exert many health-promoting effects. This study aimed to determine the effects of RGSE on serum levels of triglycerides (TG), total cholesterol (TC), high-density lipoprotein cholesterol (HDL-C), low-density lipoprotein cholesterol (LDL-C), apolipoprotein AI (apo-AI) levels and paraoxonase (PON) activity in patients with mild to moderate hyperlipidemia (MMH).

**DESIGN AND SETTINGS:**

A randomized double-blind placebo-controlled clinical trial was conducted at Shahid-Modarres Hospital (Tehran, Iran) and Tabriz University of Medical Sciences. Seventy MMH patients were randomly assigned to receive treatment (200 mg/day of RGSE) or placebo for eight weeks.

**RESULTS:**

Significant elevation in serum levels of apo-AI (P = 0.001), HDL-C (P = 0.001) and PON activity (P = 0.001) and marked decreases in concentrations of TC (P = 0.015), TG (P = 0.011) and LDL-C (P = 0.014) were found in the cases. PON activity was significantly correlated with apo-AI (r = 0.270; P < 0.01) and HDL-C (r = 0.45; P < 0.001). Significant differences between the RGSE and control groups (before and after treatment) for TC (P = 0.001), TG (P = 0.001), PON (P = 0.03), apo-AI (P = 0.001) and LDL-C (P = 0.002) were seen.

**CONCLUSION:**

It is possible that RGSE increases PON activity mostly through increasing HDL-C and apo-AI levels in MMH patients. It may thus have potential beneficial effects in preventing oxidative stress and atherosclerosis in these patients.

## RESUMO

**CONTEXTO E OBJETIVO:**

Extrato de semente de uva vermelha (RGSE) contém complexos de proantocianidinas oligoméricas como classe de flavonoides. Estes compostos são antioxidantes potentes e exercem muitos efeitos de promoção da saúde. Este estudo visou determinar os efeitos de RGSE nos níveis séricos de triglicérides (TG), colesterol total (TC), colesterol de lipoproteína alta-densidade (HDL-C), colesterol de lipoproteína baixa-densidade (LDL-C), apolipoproteína AI (apo-AI) e atividade de paraoxonase (PON) em pacientes com hiperlipidemia leve a moderada (MMH).

**DESENHO E LOCAL:**

Estudo clínico randomizado duplo-cego controlado com placebo, realizado no Hospital Shahid-Modarres (Teerã, Irã) e na Universidade de Ciências Médicas de Tabriz. Setenta pacientes com MMH foram aleatoriamente designados para receber tratamento (200 mg/dia de RGSE) ou placebo durante oito semanas.

**RESULTADOS:**

Elevação significativa nos níveis séricos de apo-AI (P = 0,001), HDL-C (P = 0,001) e atividade de PON (P = 0,001) e diminuição marcada nas concentrações de TC (P = 0,015), TG (P = 0,011) e LDL-C (P = 0,014) foram encontradas nos casos. Atividade de PON mostrou correlação significativa com apo-AI (r = 0,270; P < 0,01) e HDL-C (r = 0,45; P < 0,001). Diferenças significativas entre os grupos RGSE e controle (antes e após tratamento) para TC (P = 0,001), TG (P = 0,001), PON (P = 0,03), apo-AI (P = 0,001) e LDL-C (P = 0,002) foram observadas.

**CONCLUSÃO:**

É possível que RGSE aumente atividade de PON principalmente através da elevação dos níveis de HDL-C e apo-AI em pacientes MMH. Ele pode, assim, ter efeitos benéficos potenciais na prevenção de estresse oxidativo e aterosclerose nesses pacientes.

## INTRODUCTION

Hyperlipidemia and serum lipoprotein disorders have long been known as risk factors for progression of atherosclerosis and cardiovascular disease. Lipoprotein deposition in the intimal layer of arteries causes formation of atherosclerotic plaque.^1^^,^^2^^,^^3^ Changes to serum lipid profile can induce formation of hydroperoxide and lysis of phospholipids, oxysterol and other lipids.^1^ Oxidation of low-density lipoprotein cholesterol (LDL-C) in the arterial walls is accepted as an important mechanism for atherosclerosis. Many studies have focused on preventing LDL-C oxidation mechanisms.^4^ It has also been shown that serum levels of high-density lipoprotein cholesterol (HDL-C) and apolipoprotein-AI (apo-AI) are lower in atherosclerotic patients than in the healthy population. Decreased HDL-C levels also have an important role in increasing the risk of cardiovascular diseases.^5^ Human paraoxonase (PON) is a calcium-dependent enzyme (hydrolase) with 354 amino acids and molecular weight of 43 kDa, which is produced by the liver and released into serum. It is mainly associated with the apo-AI that is located in HDL-C. *In vitro* experiments have shown that PON can inhibit procreation of oxidized LDL (ox-LDL).^6^^,^^7^ Because of the key role of LDL-C oxidation in inducing atherosclerosis, reduced serum activity of PON may explain one of the essential mechanisms for increased risk of atherosclerosis and cardiovascular disease in hyperlipidemic patients.^8^ Moreover, it has been shown that PON prevents peroxidation of cholesterol esters.^9^ Therefore, interventions that can increase apo-AI levels, PON activity and HDL-C levels may decrease the progression of atherosclerosis.

Red grape seed extract (RGSE) contains oligomeric pro-anthocyanidin complexes (OPCs) as a class of flavonoids. These compounds are potent antioxidants and exert many health-promoting effects.^10^ The antioxidant effect of OPCs is approximately 50 times greater than that of vitamin C and vitamin E.^11^ The impact of RGSE on atherosclerosis has been studied, and some beneficial effects of these compounds against atherosclerosis have been reported.^12^^,^^13^^,^^14^

## OBJECTIVE

Because only limited information about the effects of RGSE on PON activity and on its relationships with apo-AI and HDL-C levels is available, this study was carried out to determine the effects of RGSE supplementation on the lipid profile, apo-AI levels and PON activity in patients with mild to moderate hyperlipidemia (MMH).

## METHODS

A randomized double-blind placebo-controlled clinical trial was carried out at Shahid-Modarres Hospital (Tehran, Iran) and at Tabriz University of Medical Sciences. The target population was adults aged 21-64 years, and individuals with MMH (triglycerides, TG > 150 mg/dl; and total cholesterol, TC > 200 mg/dl) were included in this study. Individuals with severe hyperlipidemia (TG > 300 mg/dl; TC > 250 mg/dl), diabetes mellitus, severe and/or poorly controlled hypertension (except mild hypertensive patients with acceptable blood pressure (BP) control through a low-salt diet alone), body mass index (BMI) > 30 kg/m^2^, heart failure, chronic renal failure, chronic hepatic disease, malignancy, any lipid-lowering drug use, vegetarian diet, alcohol use and cigarette use were excluded from the study.

All the participants signed an informed consent and the study was registered in ClinicalTrials.gov (IRCT ID: IRCT138902073812N1). The grape seed capsules used in this study were prepared at the Drug Applied Research Center (Tabriz, Iran). The capsule ingredients were as follows: dicalcium phosphate, gelatin, microcrystalline cellulose and 100 mg of RGSE. Each capsule contained the equivalent of 5-8 grape seeds. In an analysis performed on these capsules, they were found to contain at least 95% proanthocyanidins and 80% other polyphenolic compounds.

The individuals thus recruited participated in a series of joint meetings to become acquainted with the aims and significance of the study. The participants were advised not to change their lifestyle, general nutritional habits or daily physical activity during the study period and were kept under observation throughout the study period. They were encouraged to continue consuming any dietary supplementation or medication that they were using before the study, but were asked to stop consumption of any grape product during the active phases. Informed consent was obtained from all patients after they had been given explanations about the stages and aims of the study. The study was approved by the ethics committee of Tabriz University.

The RGSE group received capsules containing 200 mg/day of RGSE for eight weeks, and the placebo group received similar-looking capsules (filled with starch and cellulose) for the same amount of time. The physical activity levels of the patients were assessed using the short form of the international physical activity questionnaire and daily nutritional intake was recorded using a food frequency questionnaire at the beginning and end of each round. The questionnaires allowed the interviewer to calculate the amount of each dietary intake category (protein, carbohydrate and fat), expressed as grams/day.

Initial blood samples were taken after overnight fasting at the beginning of the study and a second blood sample was collected at the end of the round. We collected venous blood samples of 10 ml after 12 hours of fasting at the beginning of the study and after eight weeks of treatment. The lipid parameters were determined from the fresh serum samples and these samples were then stored at -70 °C for PON activity measurement before the biochemical analysis. The serum levels of apo-AI were assayed by means of commercially available immunoturbidimetric kits. Serum PON activity was determined spectrophotometrically using paraoxon (O,O-diethyl-O-P nitrophenylphosphate) as the substrate. TC, TG, and HDL-C levels were determined using commercial kits (Pars Azmoon, Tehran, Iran) with an automated chemical analyzer (Hitachi 917).

Sample size calculations were performed using the Power and Sample Size Calculation (PS) software, version 3.1.2. We studied a continuous-response variable from independent control and experimental subjects, with one placebo subject per RGSE subject. The calculation was based on information obtained from a pilot study, taking the response within each normally distributed subject group to be a true difference in the RGSE and control means of up to five subjects. Thus, the sample size was estimated to be nearly 30 subjects per group, in order to achieve an alpha of 5% and a power of 80%. The dropout rate was taken to be 15% and therefore the sample size was increased to 35 in each group. The SPSS 18 for Windows software was used to perform statistical analyses. We performed a per-protocol analysis. Results are presented as mean values ± standard deviation (SD). After determining the distribution of continuous variables using the Kolmogorov-Smirnov test, the paired Student’s t test was used to assess the significance of intra-group changes during the intervention period and an independent-sample t test was applied to compare the results of the two groups. Correlations were evaluated using Pearson’s test and the statistical significance level was set at P < 0.05.

## RESULTS

Seventy-five patients were initially recruited. Five patients failed to complete the study period because of lack of cooperation and the analysis was performed on a total of 70 cases (43 females). The mean age of the subjects was 48.22 ± 9.07 years.

Although the daily protein and carbohydrate intake increased during RGSE intake, the mean daily fat and fiber intake did not show any meaningful change (P > 0.05) (Table 1). Data on HDL-C, LDL-C, TG, TC, body weight and systolic and diastolic blood pressures are shown in Table 2. The placebo and RGSE groups did not show any significant differences in the means of any of the variables at the beginning of the study (Table 2). The mean lipid profile, apo-AI levels and PON activity in the RGSE and control group after eight weeks are shown in Table 2. Significant differences between the RGSE and control groups (both before and after the treatment) were seen in relation to TC (P = 0.001), TG (P = 0.001), PON (P = 0.03), apo-AI (P = 0.001) and LDL-C (P = 0.002) (Table 2).

**Table 1.**
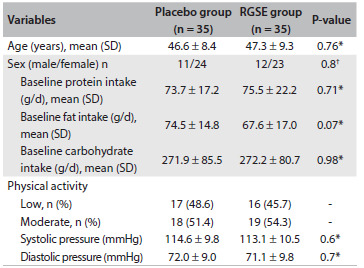
Characteristics of study groups at baseline

RGSE = red grape seed extract; SD = standard deviation. *Performed using independent-sample t test; ^†^Performed using chi-square test.

**Table 2.**
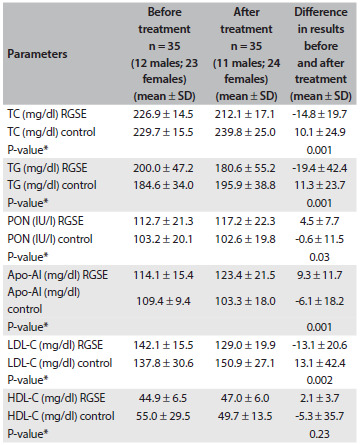
Lipid profile, apo-AI levels and PON activity in the RGSE and control groups

apo-AI = apolipoprotein AI; PON = paraoxonase; RGSE = red grape seed extract; SD = standard deviation; TC = total cholesterol; TG = triglyceride; HDL = high-density lipoprotein; LDL = low-density lipoprotein. Values are reported as mean ± SD. *Comparison of the differences (before and after treatment) between RGSE and control groups (independent-sample t test).

To determine the effects of RGSE and placebo on PON activity, the correlation between the degree of change in PON after two months (Δ PON) with the degrees of change in HDL-C (Δ HDL-C) and in apo-AI (Δ apo-AI) and also the correlation between Δ HDL-C and Δ apo-AI are shown in Table 3. Significant correlations between Δ PON and Δ HDL-C (P < 0.001; r = 0.672) and between Δ PON and Δ apo-AI (P < 0.01; r = 0.427) were seen in the RGSE group. In addition, Δ apo-AI significantly correlated with Δ HDL-C (P < 0.05; r = 0.408) in the RGSE group.

**Table 3.**
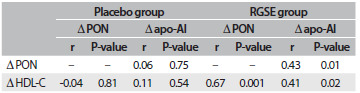
Correlations between the changes in PON activity, HDL-C and apo-AI levels after two months of RGSE (red grape seed extract) or placebo treatment

PON = paraoxonase; HDL-C = high-density lipoprotein cholesterol, apo-AI = apolipoprotein AI; Δ PON = PON (after two months of RGSE or placebo treatment) minus PON (baseline); Δ HDL-C = HDL-C (after two months of RGSE or placebo treatment) minus HDL-C (baseline); Δ apo-AI = apo-AI (after two months of RGSE or placebo treatment) minus apo-AI (baseline); Pearson’s test was used to determine correlations between Δ PON, Δ apo-AI and Δ HDL-C.

## DISCUSSION

Clinical and epidemiological studies have over the years established that dyslipidemia constitutes the main risk factor for atherosclerosis.^15^ Although hyperlipidemia is a major factor for atherosclerosis, lipid peroxidation also has an important role in this process.^16^ Oxidative damage plays a key role in accelerated atherosclerosis and is involved in cardiovascular disease among hyperlipidemia patients who are at risk of increased oxidative stress.^17^ There is a growing body of evidence demonstrating that reduced activity of the HDL-associated enzyme PON is predictive of vascular disease in humans. This evidence includes the results from prospective studies.^18^ It has been suggested that some drugs with lipid-lowering properties can alter lipid peroxidation products.^19^^,^^20^ Unlike drugs, which are associated with some toxic effects, natural antioxidants have beneficial effects.^21^^,^^22^^,^^23^ RGSE, which is now available as a dietary supplement, contains a number of polyphenols, including procyanidins and proanthocyanidins, which are powerful free radical scavengers.^24^^,^^25^ The exact chemical characteristics and the mechanism of action of RGSE have not yet been completely understood and the experimental findings are inconsistent.

It has been demonstrated that serum concentrations of apo-AI are a better indicator of coronary heart disease (CHD) than serum lipid and lipoprotein levels.^26^^,^^27^ The results from this study indicate that RGSE administration can significantly increase the apo-AI levels in MMH patients. Some studies, such as those by Ignea et al.^28^ and El-Alfy et al.,^29^ showed that RGSE can increase the activity of antioxidant enzymes and can prevent lipid peroxidation. In this study, we showed that two months of RGSE administration can lead to increased PON activity in MMH patients. Furthermore, we previously showed that RGSE administration can improve lipid profiles and lead to decreased ox-LDL.^30^ Human PON is a calcium-dependent esterase closely associated with HDL. It contains apo-AI, which has been reported to confer antioxidant properties on HDL-C through decreasing the accumulation of lipid peroxidation products.^31^^,^^32^ HDL-C protects against atherosclerosis by returning excess cholesterol from peripheral tissues back to the liver for reuse or excretion into the bile. Several reports have suggested that HDL may have an antioxidative function, which may contribute towards its anti-atherogenic activity.^33^^,^^34^^,^^35^

In the study by Song et al.,^36^ 28 days of administration of grape seed powder was found to be capable of reducing the levels of serum lipids (TC, TG and LDL-C) and preventing occurrences of fatty liver among rats. In confirmation of this study, we demonstrated that RGSE had the capacity to significantly increase the concentration of HDL-C apo-AI and lead to decreased TC, TG and LDL-C levels, in relation to the pre-treatment values. Moreover, significant correlations of PON with both apo-AI and HDL-C were found. A significant correlation between the changes in HDL-C and apo-AI levels following two months of RGSE administration was also noticed.

The major limitations of the present study included the short duration of the study, the use of only a single dosage of RGSE and the lack of measurements on the levels of apo-J, superoxide dismutase, catalase, glutathione peroxidase activity and antioxidant capacity. However, to the best of our knowledge, this was the first study to attempt to show the effect of RGSE on PON in people with MMH.

## CONCLUSIONS

It seems that RGSE administration increases PON activity through increasing the HDL-C and apo-A1 levels and/or cooperative increases in the concentrations of both factors in MMH patients. In conclusion, RGSE administration in MMH patients has beneficial effects on the lipid and apolipoprotein profiles. It increases PON activity mostly through increasing HDL-C and apo-AI levels. It may have potential beneficial effects on oxidative stress and can exert an anti-atherosclerotic effect in MMH patients, which is effected by increased PON activity. Studies involving a larger population size should be conducted in order to confirm these hypotheses.
